# A dataset of 352 nuclear genes for accurate species identification and geographical origin traceability of *Rhododendron dauricum* L

**DOI:** 10.1016/j.dib.2026.112911

**Published:** 2026-06-04

**Authors:** Xiaoyi Cheng, Dan Wang, Kai Mu, Chao Xu, Jin Zhang, Xueying Yang, Jie Zhang

**Affiliations:** a College of Landscape Architecture, Northeast Forestry University, Harbin 150040, China; b Engineering Research Centre of Forestry Biotechnology of Jilin Province, College of Forestry, Beihua University, Jilin 132013, China; c Key Laboratory of Systematic and Evolutionary Botany/ State Key Laboratory of Plant Diversity and Specialty Crops, Institute of Botany, Chinese Academy of Sciences, Beijing 100093, China; d China National Botanical Garden, Beijing 100093, China; e National Engineering Laboratory for Forensic Science, Key Laboratory of Forensic Genetics, Institute of Forensic Science, Ministry of Public Security, Beijing 100038, China

**Keywords:** *Rhododendron dauricum*, Whole genome skimming, Nuclear markers, Species identification, Geographical origin traceability

## Abstract

This dataset presents 352 nuclear genes assembled from whole genome skimming data of 43 *Rhododendron* samples. The data were generated from 14 *Rhododendron dauricum* collected from seven distinct geographical populations in Northeast China, together with sequence data from 29 additional *Rhododendron* samples downloaded from the NCBI database. Using the universal set of 353 angiosperm nuclear genes as a reference, all genes were assembled with the HybPiper v2.1.1 pipeline. The dataset contains raw assembly sequences in FASTA format for each gene. Sequence alignment, trimming, and phylogenetic analysis were performed to construct phylogenetic trees. The resulting phylogenies based on concatenated 352-gene dataset and the screened 17-gene sub-dataset clearly distinguished *R. dauricum* from other *Rhododendron* species. Moreover, both datasets resolved individuals from the same population into distinct clades, enabling geographical origin traceability for the protected species *R. dauricum*. This dataset provides high-resolution molecular markers for research on *Rhododendron* phylogenomics, population genetics, conservation, and molecular identification.

Specifications Table

**Table:** 

Subject	Biology
Specific subject area	Bioinformatics, Genomics
Type of data	Tables, Figures. Raw, Analyzed.
Data collection	Leaves of 14 *Rhododendron dauricum* L. individuals from seven populations in Northeast China were sampled, and silica-dried for total genomic DNA extraction using modified CTAB method. Whole genome skimming (WGS) was performed on DNBSEQ-T7 sequencer. Additional 29 *Rhododendron* WGS data were downloaded from NCBI SRA database. Software Fastp v0.23.2 was used for sequencing data quality control, HybPiper v2.1.1 for 353 nuclear coding sequence capture, MAFFT v7.505 for sequence alignment, trimAl v1.4.rev22 for alignment trimming, ModelFinder Plus for optimal model screening of sequence matrix, IQ-TREE v2.2.0 for phylogenetic tree construction, MEGA v12 for site statistics, DnaSP v6.12.03 for Pi value inference, and R package for K2P genetic distance and PCoA analysis.
Data source location	*Rhododendron dauricum* materials were collected from seven distinct natural populations in Northeast China: XK: Xingkai Town, Mishan City, Heilongjiang Province; YH: Youhao District, Yichun City, Heilongjiang Province; JX: Jinshantun District, Yichun City, Heilongjiang Province; TH: Tahe County, Daxing'anling Prefecture, Heilongjiang Province; MX: Meixi District, Yichun City, Heilongjiang Province; XA: Fangzheng County, Harbin City, Heilongjiang Province; MO: Moerdaoga Town, Hulunbuir City, Inner Mongolia Autonomous Region.
Data accessibility	Repository name: NCBI. The NCBI links to the sequenced data can be accessed at: https://www.ncbi.nlm.nih.gov/bioproject/1428513 https://www.ncbi.nlm.nih.gov/biosample/SAMN55955831 https://www.ncbi.nlm.nih.gov/biosample/SAMN55955832 https://www.ncbi.nlm.nih.gov/biosample/SAMN55955833
	https://www.ncbi.nlm.nih.gov/biosample/SAMN55955834 https://www.ncbi.nlm.nih.gov/biosample/SAMN55955835 https://www.ncbi.nlm.nih.gov/biosample/SAMN55955836 https://www.ncbi.nlm.nih.gov/biosample/SAMN55955837 https://www.ncbi.nlm.nih.gov/biosample/SAMN55955838 https://www.ncbi.nlm.nih.gov/biosample/SAMN55955839 https://www.ncbi.nlm.nih.gov/biosample/SAMN55955840 https://www.ncbi.nlm.nih.gov/biosample/SAMN55955841 https://www.ncbi.nlm.nih.gov/biosample/SAMN55955842 https://www.ncbi.nlm.nih.gov/biosample/SAMN55955843 https://www.ncbi.nlm.nih.gov/biosample/SAMN55955844 https://www.ncbi.nlm.nih.gov/sra/?term=SRR37381074 https://www.ncbi.nlm.nih.gov/sra/?term=SRR37381073 https://www.ncbi.nlm.nih.gov/sra/?term=SRR37381068 https://www.ncbi.nlm.nih.gov/sra/?term=SRR37381067 https://www.ncbi.nlm.nih.gov/sra/?term=SRR37381066 https://www.ncbi.nlm.nih.gov/sra/?term=SRR37381065 https://www.ncbi.nlm.nih.gov/sra/?term=SRR37381064 https://www.ncbi.nlm.nih.gov/sra/?term=SRR37381063 https://www.ncbi.nlm.nih.gov/sra/?term=SRR37381062 https://www.ncbi.nlm.nih.gov/sra/?term=SRR37381061 https://www.ncbi.nlm.nih.gov/sra/?term=SRR37381072 https://www.ncbi.nlm.nih.gov/sra/?term=SRR37381071 https://www.ncbi.nlm.nih.gov/sra/?term=SRR37381070 https://www.ncbi.nlm.nih.gov/sra/?term=SRR37381069 Repository name: Science Data Bank. The Science Data Bank links to the analyzed data can be accessed at: 10.57760/sciencedb.30507
Related research article	None.

## 1 Value of the Data

• This dataset provides 352 assembled nuclear gene sequences for *Rhododendron dauricum* and related species, filling a critical gap in genomic resources for this ecologically and economically important genus. It offers high-resolution molecular markers that overcome the limitations of traditional chloroplast markers, enabling robust phylogenetic and population genetic analyses.
• From the 352 genes, a sub-dataset of 17 highly informative nuclear genes was identified, providing a cost-effective tool for species identification and geographical origin traceability. This mini-core marker set is particularly valuable for monitoring illegal harvesting, verifying germplasm provenance, and supporting conservation management of the protected species *R. dauricum*.
• Phylogenetic and population genetic analyses validated the dataset’s effectiveness, clearly resolving individuals from different geographical populations into distinct clades and showing lower within-population than between-population genetic distances. These results highlight the dataset’s utility for accurate population discrimination and origin authentication, with broader applicability to other threatened plant species.

## 2 Background

**Fig. 1:**
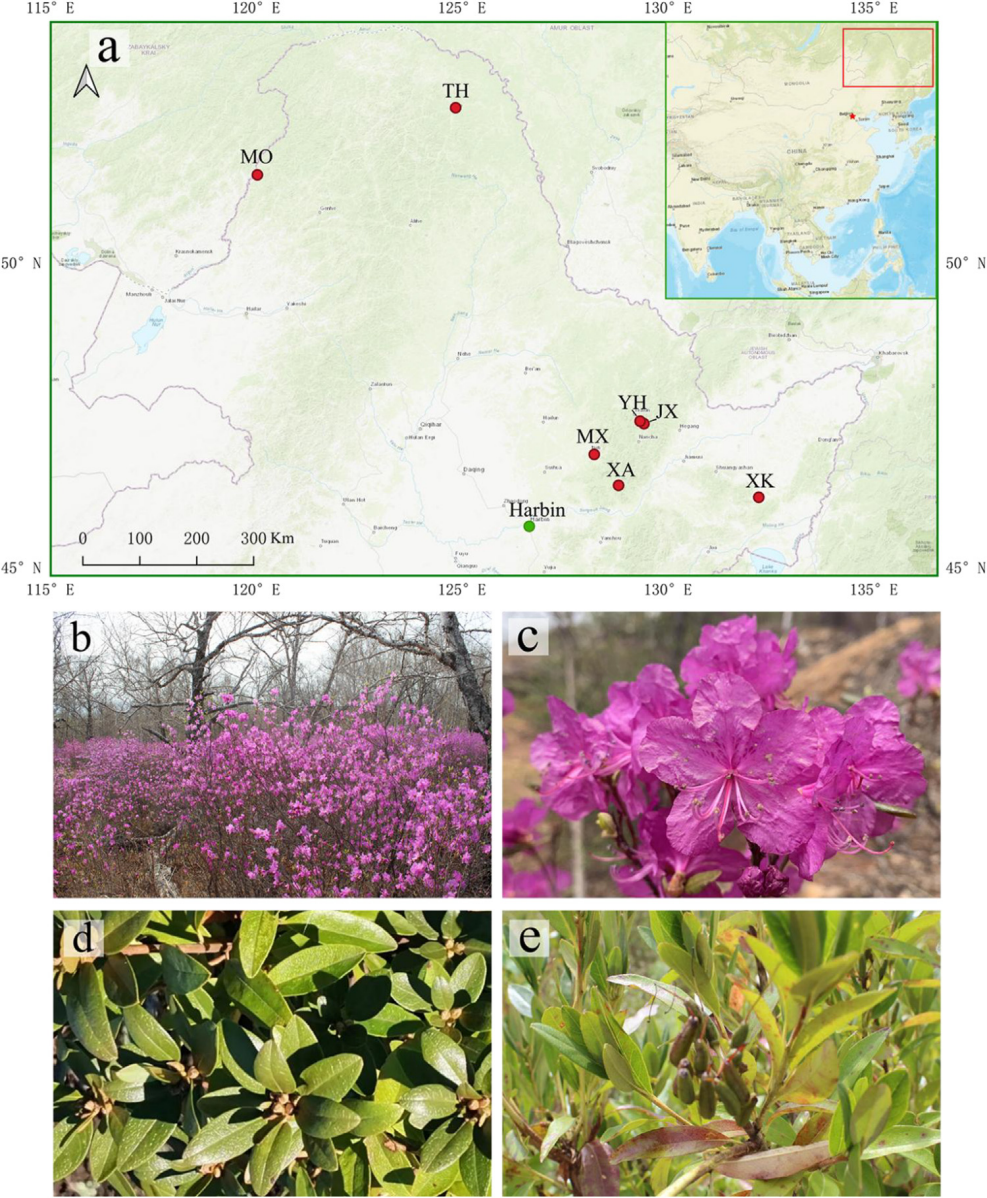
Sampling locations and morphological characteristics of *Rhododendron dauricum*. (a) Geographical distribution of the seven sampled populations in Northeast China (red dots). Population codes: XA, MO, YH, JX, TH, MX, XK. (b–e) Morphological characteristics of *Rhododendron dauricum*: (b) Morphology and habitat of the *Rhododendron dauricum,* (c) flower, (d) leaf, (e) fruit (photo by Dan Wang).

*Rhododendron dauricum* L. (Ericaceae) is a deciduous shrub widely distributed across Northeast China, Siberia, and the Russian Far East (Fig. 1). It plays important ecological roles in cold-temperate forests, contributing to understory structure, soil stabilization, and spring nectar resources for pollinators. In addition to its ecological significance, the species holds considerable horticultural value due to its early flowering and ornamental traits (Fig. 1), and has been used in traditional medicine in some regions. Owing to increasing anthropogenic pressures, including overharvesting for ornamental and medicinal purposes, as well as habitat fragmentation, *R. dauricum* was listed as a nationally protected wild plant (Level II) in China in 2021 [1].

Previous population genetic studies of this species have relied primarily on chloroplast DNA markers, which provide limited resolution for distinguishing closely related populations and individuals. While chloroplast-based analyses have revealed spatial genetic structure shaped by Quaternary climate changes [2,3], nuclear markers which offer higher resolution for species identification and population discrimination remain scarce. Unlike uniparentally inherited chloroplast markers, nuclear genes provide biparental inheritance and higher polymorphism, making them more suitable for resolving close relationships. The Angiosperms 353 probe set, designed by the Royal Botanic Gardens, Kew, targets 353 highly conserved nuclear genes across flowering plants [4]. This approach enables simultaneous recovery of hundreds of nuclear loci, facilitating phylogenomic studies and the development of mini-core marker sets for practical applications.

Nuclear gene markers have been successfully applied in species identification and geographical traceability. In Eastern Mediterranean orchids, Angiosperms 353 distinguished over 50 species and identified 30 highly informative loci for DNA barcoding [5]. A study on the endangered Chinese endemic *Cathaya argyrophylla* developed transcriptome-derived SNP markers to trace geographical origin, identifying 113 population-specific SNPs from 69 individuals across five populations [6]. This approach demonstrates the feasibility of using nuclear genetic markers for geographical traceability of threatened plant species.

Recent advances in genome skimming have demonstrated the feasibility of recovering hundreds of nuclear genes from herbarium material, with approximately 15× sequencing depth representing a cost-effective strategy for phylogenomic studies in *Rhododendron* [7]. In this study, we present a dataset of 352 nuclear gene sequences from *R. dauricum* and related species. This dataset and related method offer a cost-effective solution for forensic applications, including monitoring illegal harvesting and verifying germplasm provenance of *R. dauricum*. Such strategies can be readily adapted for other protected species requiring geographic origin authentication.

## 3 Data Description

This dataset comprises coding sequences (CDS) of 352 nuclear genes retrieved from 43 *Rhododendron* samples using the 353 universal Angiosperms nuclear gene set [4] as reference. Based on recovery rates, 352 genes were retained for downstream analyses. The dataset covers 14 *Rhododendron dauricum* individuals collected from seven populations in Northeast China and 29 other *Rhododendron* samples whose whole genome skimming data were downloaded from the NCBI SRA database. Detailed sample information is provided in Supplementary Table S1.

Gene recovery statistics are provided in Supplementary Table S2. The recovery rate for the 14 *Rhododendron dauricum* samples is 100% across the 352 target genes. For the 29 NCBI-downloaded samples, the recovery rate ranges from 90.34% to 100%, with an average of 97.84%. In terms of sequence completeness, the number of complete genes (≥500 bp) across all samples ranges from 198 to 231, with an average proportion of complete sequences of 65.00%. For the percentage of target genes recovered with ≥70% of the reference length, the 14 *R. dauricum* samples range from 99.43% to 99.72%, while the 29 NCBI-downloaded samples range from 92.79% to 100%, with an overall mean of 98.57% across all 43 samples. Gene recovery patterns across all 43 samples are visualized in a heatmap (Supplementary Fig. S1), where the x-axis represents the 352 target genes, the y-axis represents the 43 samples, and the color intensity of each cell indicates the completeness of the recovered gene for that sample.

The numbers of Constant Sites, Variable Sites, Parsimony-informative Sites, Singleton Sites, and the nucleotide diversity (Pi, π) values, as well as average coverage breadth, were provided for 352 genes in Supplementary Table S3. Based on the counts of parsimony-informative sites, the 17 most informative genes were selected as a subset from the 352 genes and adopted as the core marker set for population discrimination. The selection threshold of >100 parsimony-informative sites was established to capture the most phylogenetically informative markers while maintaining practical utility for population-level analysis (see Supplementary Fig. S4 for distribution of parsimony-informative sites across all 352 genes). Genes below this threshold contribute limited phylogenetic signal and tend to produce poorly resolved or unstable topologies in preliminary analyses. For these 17 genes, the number of parsimony-informative sites ranges from 101 to 220, with an average of 124.8 per gene.

**Fig. 2:**
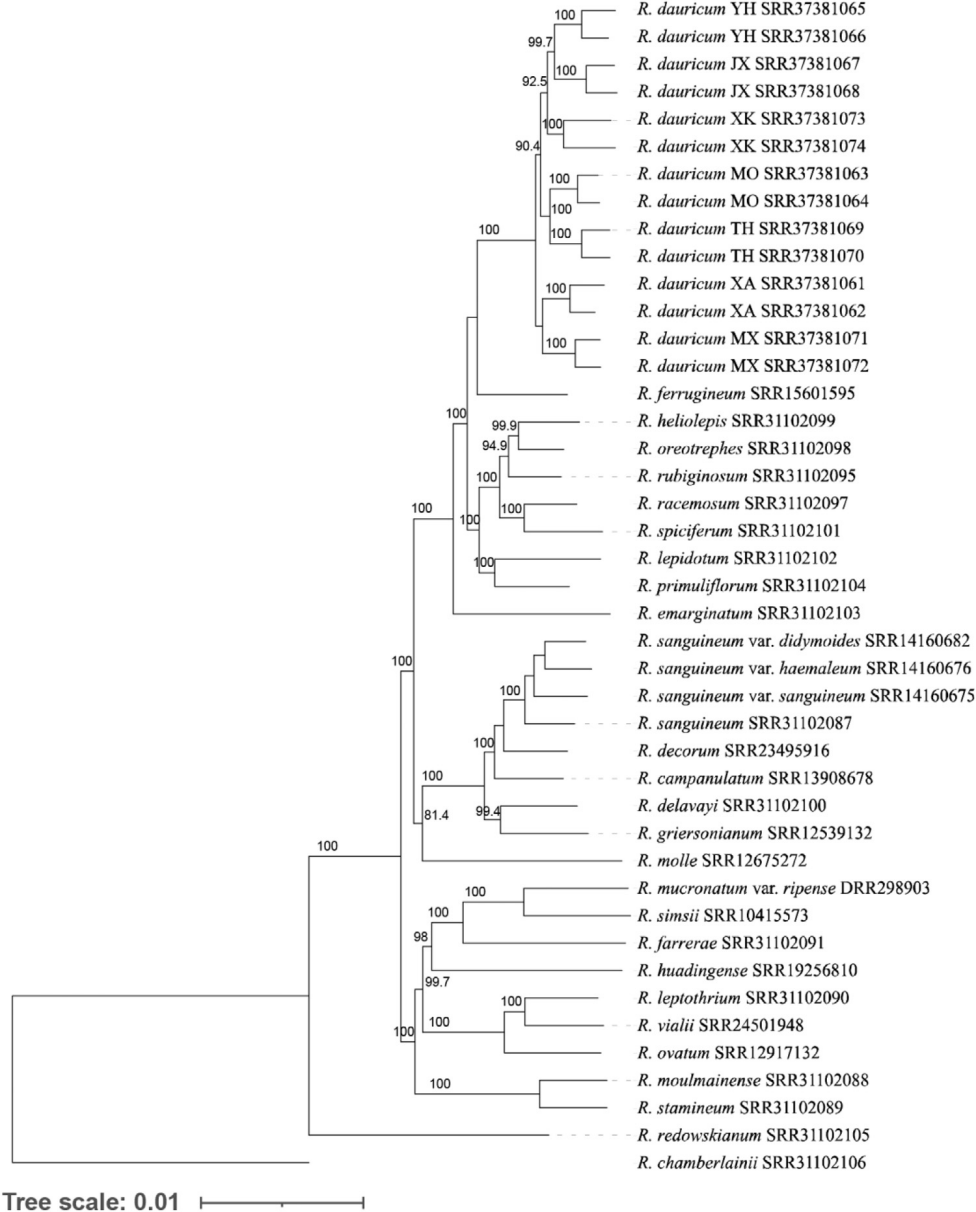
Maximum likelihood trees constructed based on 352 nuclear genes. Numbers on branches indicate ultrafast bootstrap support (UFBoot, 1000 replicates). A scale bar represents the number of substitutions per site.

**Fig. 3:**
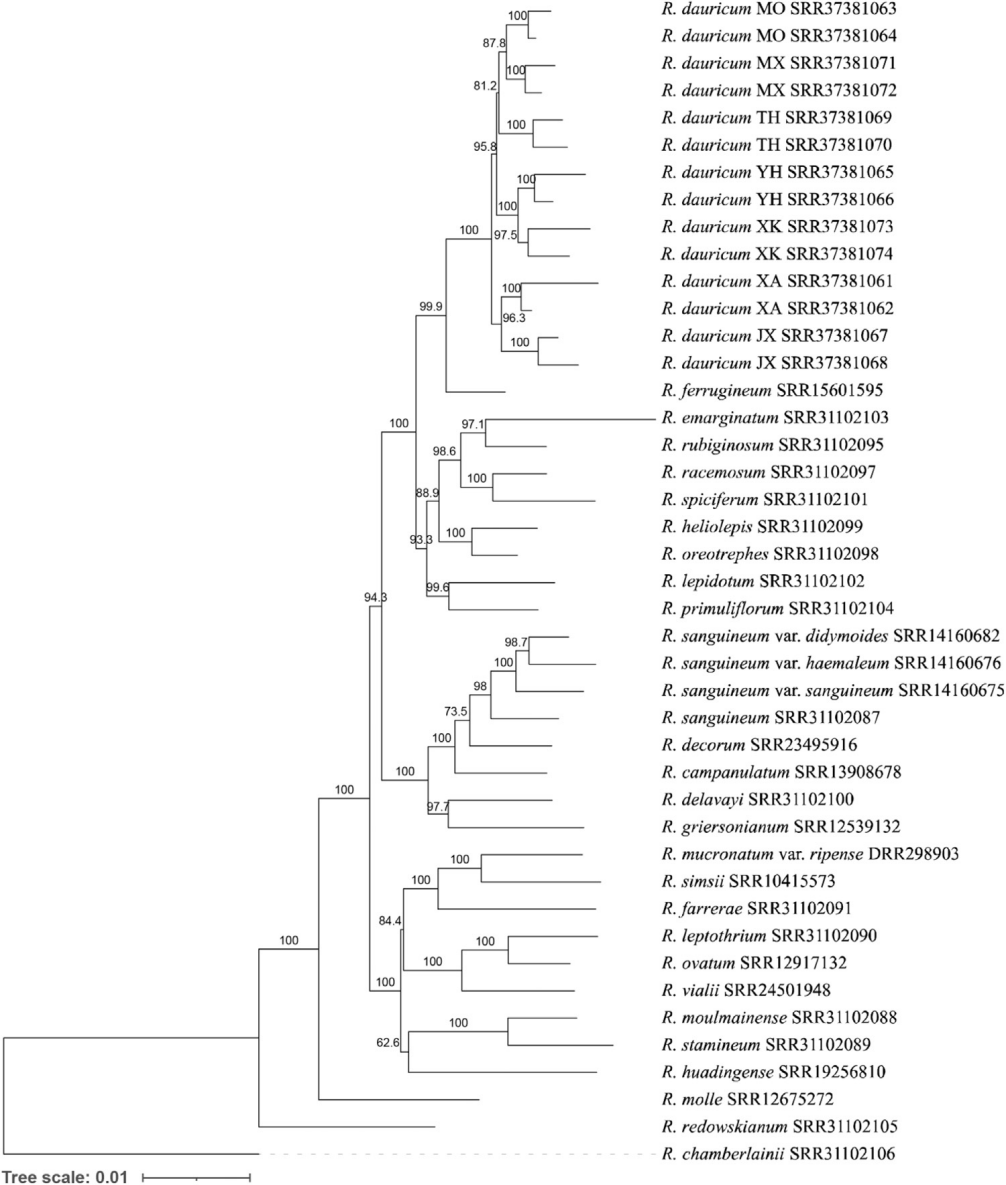
Maximum likelihood trees constructed based on 17 nuclear genes with the highest number of parsimony-informative sites. Numbers on branches indicate ultrafast bootstrap support (UFBoot, 1000 replicates). A scale bar represents the number of substitutions per site.

This study constructed a phylogenetic tree using the maximum likelihood method, which clearly demonstrates the branching relationships between *Rhododendron dauricum* and other related species (Fig. 2). In the phylogenetic tree, *Rhododendron dauricum* was located on an independent branch of the *Rhododendron* genus with 100 % bootstrap support, forming a sister group relationship with *Rhododendron ferrugineum*. To further verify the accuracy of the core marker set for population discrimination, we conducted a comparative analysis using the 17 informative genes (Fig. 3), and the results showed that the main branch relationships remained consistent, both successfully resolving the 14 *Rhododendron dauricum* individuals from the seven populations into their respective groups.

To validate the superior resolution of the 352-gene nuclear dataset, we also constructed phylogenetic trees using three traditional DNA barcoding markers—rbcL, matK (chloroplast DNA), and ITS (nuclear ribosomal DNA)—as shown in Supplementary Fig. S3. Compared to the 352-gene tree (Fig. 2) and the 17-gene tree (Fig. 3), the traditional markers produced substantially lower resolution. Notably, the ITS-based tree failed to recover *R. dauricum* as a monophyletic group, and neither traditional markers resolved all individuals from different geographical populations into distinct clades. These comparisons demonstrate the superior resolution of the nuclear gene markers developed in this study.

**Fig. 4:**
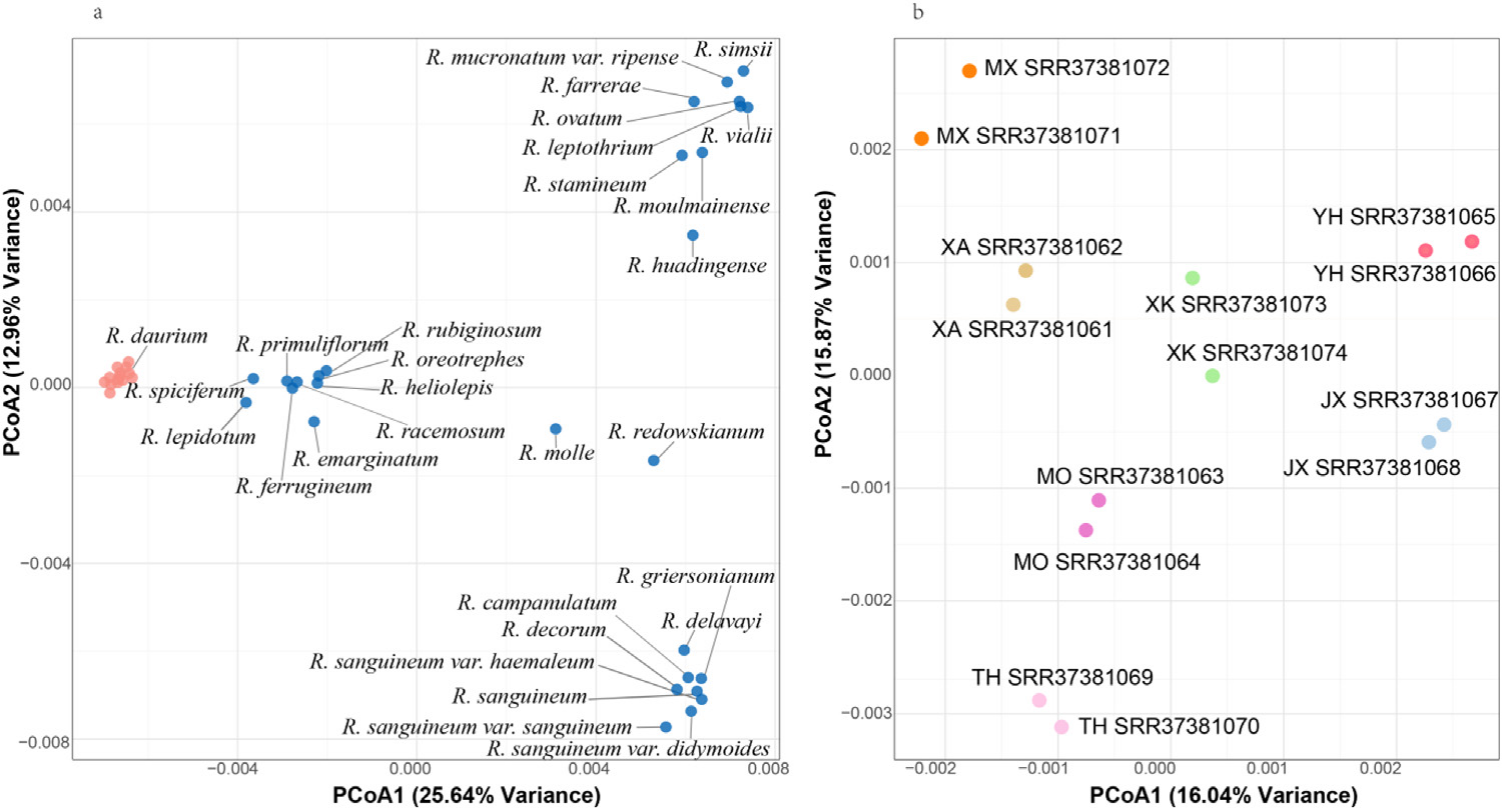
Principal Coordinate Analysis (PCoA) plots based on K2P genetic distances. (a) PCoA plot of 42 analyzed *Rhododendron* samples, showing clustering patterns consistent with their phylogenetic relationships. The first and second principal coordinates explain 25.64% and 12.96% of the total genetic variation, respectively. *Rhododendron dauricum* samples were colored in red, the others in blue. (b) PCoA plot of 14 *R. dauricum* individuals from seven populations in Northeast China. The two individuals from each population are represented in the same color, with different populations depicted in distinct colors. The first and second principal coordinates explain 16.04% and 15.87% of the total genetic variation, respectively.

**Fig. 5:**
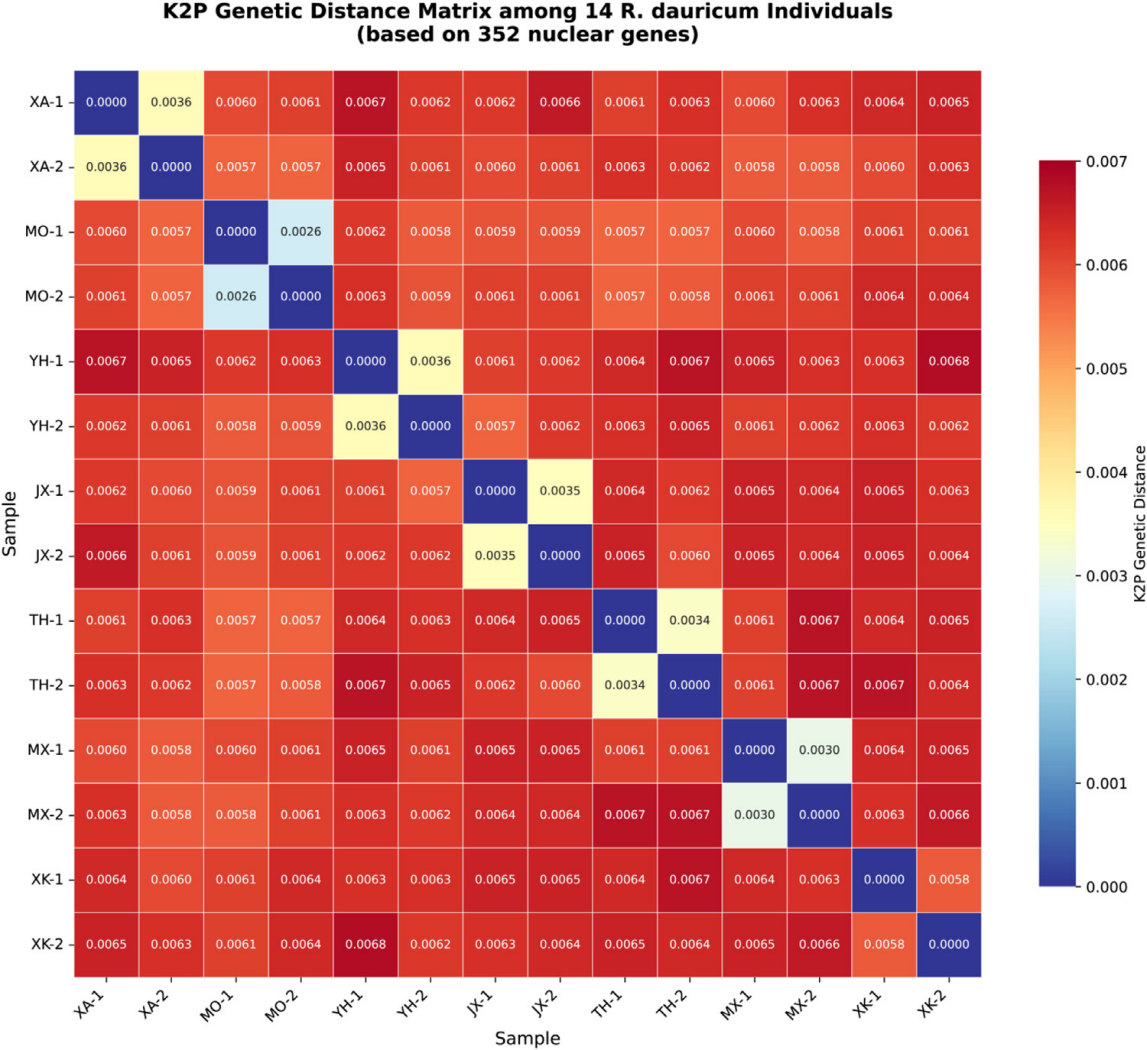
K2P genetic distance heatmap among 14 *Rhododendron dauricum* individuals based on 352 nuclear genes. Samples were collected from seven populations in Northeast China. Each population is abbreviated with two uppercase letters, and two individuals per population are denoted as 1 and 2. Warmer colors indicate larger genetic distances, and cooler colors indicate smaller genetic distances. Detailed sample information is provided in Supplementary Table S1.

Principal Coordinate Analysis (PCoA) based on K2P genetic distances further reveals the genetic differentiation patterns among samples [8]. In the PCoA plot including the 14 *Rhododendron dauricum* individuals and 28 individuals of other *Rhododendron* species (42 samples in total; Fig. 4a), the first and second principal coordinates explain 25.64% and 12.96% of the total genetic variation, respectively, with *R. dauricum* individuals clearly separated from other species in the two-dimensional space. The PCoA plot restricted to the 14 *R. dauricum* individuals (Fig. 4b) shows that the first and second principal coordinates explain 16.04% and 15.87% of the total genetic variation, with individuals from the same population cluster tightly together. The PCoA plot for all 43 samples including the outgroup is provided in Supplementary Fig. S2, as the inclusion of the *Rhododendron chamberlainii* reduced the visual resolution of intra- and inter-population relationships. Pairwise genetic distances among the 14 *Rhododendron dauricum* individuals are provided in a heatmap (Fig. 5), ranging from 0.0026 to 0.0068. In terms of genetic distance, individuals from the same population exhibit significantly smaller genetic distances compared to those from different populations.

## 4 Experimental Design, Materials and Methods

### 4.1 Data Generation and Processing

Leaf materials of 14 *Rhododendron dauricum* individuals were collected from seven populations in Northeast China, dried with silica gel, and subjected to total genomic DNA extraction using a modified CTAB method [9]. Whole genome skimming was performed on the DNBSEQ-T7 platform to obtain 150 bp paired-end reads. Raw sequencing reads were quality-filtered using fastp v0.23.2 [10] to remove low-quality bases and adapters. Additionally, 29 whole genome skimming data of other *Rhododendron* species were downloaded from the NCBI SRA database.

Using the Angiosperms353 universal nuclear gene set as a reference, coding sequences were retrieved from all 43 samples using HybPiper v2.1.1 [11]. The specific reference sequences for *Rhododendron scopulorum* and *Rhododendron tomentosum* were downloaded from the Kew Tree of Life website (https://treeoflife.kew.org). Genes with recovery rates below 50% across samples were excluded, resulting in 352 genes retained for downstream analyses. Sequences for each gene were aligned using MAFFT v7.505 [12] with the –auto parameter. Alignments were trimmed using trimAl v1.4.rev22 [13] with parameters -gt 0.8 and -st 0.001, retaining columns present in at least 80% of sequences (with gaps treated as missing) and further filtering based on a similarity score threshold of 0.001, followed by a sliding-window approach (window size = 10 bp, step size = 5 bp) to remove highly variable regions when the sequence similarity within that window fell below 70%.

### 4.2 Phylogenetic Analysis

Individual gene alignments were concatenated using AMASv1.0.6 [14] to generate a concatenated supermatrix. Phylogenetic reconstruction was performed on the concatenated dataset using IQ-TREE v2.2.0 [15] with a partitioned model. The optimal nucleotide substitution model was automatically selected using ModelFinder Plus based on the Bayesian Information Criterion. Nodal support was assessed using ultrafast bootstrap with 1,000 replicates.

To identify a core marker set for population discrimination, the number of parsimony-informative sites for each of the 352 genes was calculated using MEGA v12 [16], and nucleotide diversity (Pi) was estimated using DnaSP v6.12.03 [17]. The genes were ranked in descending order based on the number of parsimony-informative sites (Table S3), and the 17 genes with the number value over 100 were selected. Following the same phylogenetic workflow described above, a second tree was constructed using the concatenated 17-gene sub-dataset. To further compare with the resolution of the 17-gene set, we constructed phylogenetic trees using three traditional markers (rbcL, matK, and ITS). Reference sequences were obtained from GenBank: the complete chloroplast genome of *Rhododendron aureum* (OM574981) for extracting rbcL and matK, and the ITS sequence of *Rhododendron kanehirae* (AF172290) for ITS. Using the same assembly, alignment, trimming, and phylogenetic reconstruction procedures as described above for the nuclear genes, these markers were processed and the resulting trees are presented in Supplementary Fig. S3. The assembled sequences of rbcL, matK, and ITS have also been deposited in the publicly accessible repository ScienceDB (10.57760/sciencedb.30507).

### 4.3 Genetic Distance and PCoA Analysis

Genetic distances among the 14 *Rhododendron dauricum* individuals were calculated based on the concatenated matrix of 352 nuclear genes using the ape package v5.7 in R v4.5.1. The Kimura 2-parameter (K2P) model was applied, with pairwise deletion for missing sites.

Principal Coordinate Analysis (PCoA) was performed on the K2P genetic distance matrices using the “ cmdscale ” function in R to reduce the distance matrix to two dimensions for visualization. The PCoA plots were drawn for the 42 *Rhododendron* samples and the 14 *R. dauricum* individuals separately. One species was excluded from the *Rhododendron* sample analysis for the purpose of revealing the finer-scale clustering patterns among the focal samples. The analysis of all 43 samples is provided in Supplementary Fig. S2.

## Limitations

None.

## Ethics Statement

All authors have read and follow the ethical requirements for publication in Data in Brief and confirming that the current work does not involve human subjects, animal experiments, or any data collected from social media platforms.

## CRediT authorship contribution statement

**Xiaoyi Cheng:** Methodology, Writing – original draft, Visualization. **Dan Wang:** Conceptualization, Investigation, Writing – review & editing. **Kai Mu:** Software, Visualization, Writing – review & editing. **Chao Xu:** Conceptualization, Data curation, Writing – review & editing. **Jin Zhang:** Funding acquisition, Supervision, Validation. **Xueying Yang:** Funding acquisition, Writing – review & editing. **Jie Zhang:** Supervision, Writing – review & editing, Project administration.

## Data Availability

Science Data BankA Dataset of 352 Nuclear Genes for Accurate Species Identification and Geographical Origin Traceability of Rhododendron dauricum L. (Original data)
NCBI BiosamplePlant sample from Rhododendron dauricum (Original data)
NCBI BiosamplePlant sample from Rhododendron dauricum (Original data)
NCBI BiosamplePlant sample from Rhododendron dauricum (Original data)
NCBI BiosamplePlant sample from Rhododendron dauricum (Original data)
NCBI BiosamplePlant sample from Rhododendron dauricum (Original data)
NCBI BiosamplePlant sample from Rhododendron dauricum (Original data)
NCBI BiosamplePlant sample from Rhododendron dauricum (Original data)
NCBI BiosamplePlant sample from Rhododendron dauricum (Original data)
NCBI BiosamplePlant sample from Rhododendron dauricum (Original data)
NCBI BiosamplePlant sample from Rhododendron dauricum (Original data)
NCBI BiosamplePlant sample from Rhododendron dauricum (Original data)
NCBI BiosamplePlant sample from Rhododendron dauricum (Original data)
NCBI BiosamplePlant sample from Rhododendron dauricum (Original data)
NCBI BiosamplePlant sample from Rhododendron dauricum (Original data)

## Appendix Supplementary materials

Image, application 1

Image, application 2

Image, application 3

Image, application 4

Image, application 5

Image, application 6

Image, application 7
