# Supplementary figures and images for: A dataset of 352 nuclear genes for accurate species identification and geographical origin traceability of Rhododendron dauricum L
Source: Data Brief. 2026 Jun 4;67:112911. doi: 10.1016/j.dib.2026.112911 (PMC13272538; doi:10.1016/j.dib.2026.112911)

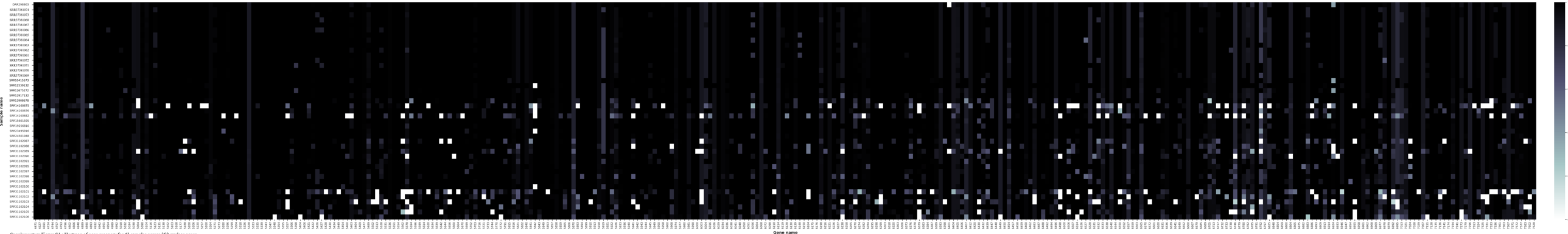

Supplement: Supplementary file 1 [file mmc1.pdf]

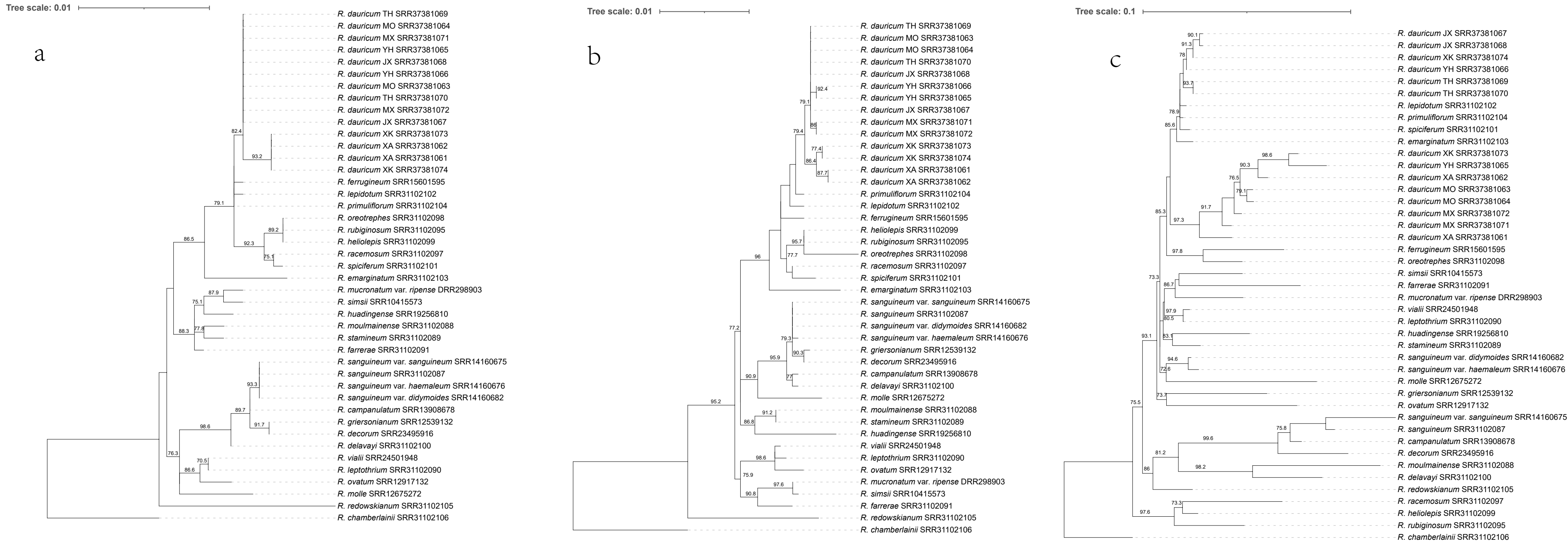

Supplement: Supplementary file 3 [file mmc3.pdf]
