# Supplementary material for: A dataset of 352 nuclear genes for accurate species identification and geographical origin traceability of Rhododendron dauricum L
Source: Data Brief. 2026 Jun 4;67:112911. doi: 10.1016/j.dib.2026.112911 (PMC13272538; doi:10.1016/j.dib.2026.112911)
Supplement: Supplementary file 2 [file mmc2.pdf]

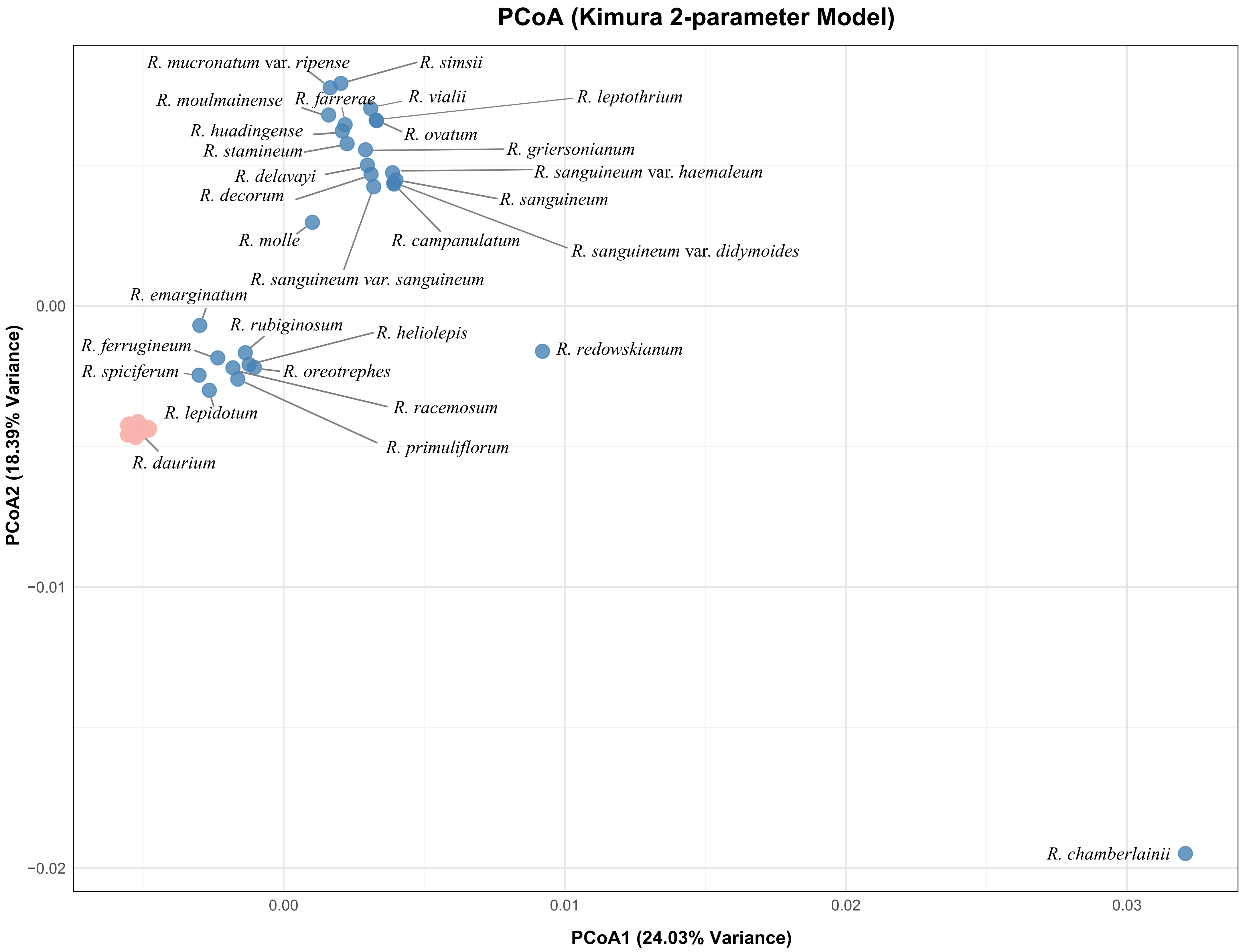

Supplementary Figure S2 : Principal Coordinate Analysis (PCoA) plot of the 43 samples based on K2P genetic distances.
