# Supplementary material for: A dataset of 352 nuclear genes for accurate species identification and geographical origin traceability of Rhododendron dauricum L
Source: Data Brief. 2026 Jun 4;67:112911. doi: 10.1016/j.dib.2026.112911 (PMC13272538; doi:10.1016/j.dib.2026.112911)
Supplement: Supplementary file 4 [file mmc4.pdf]

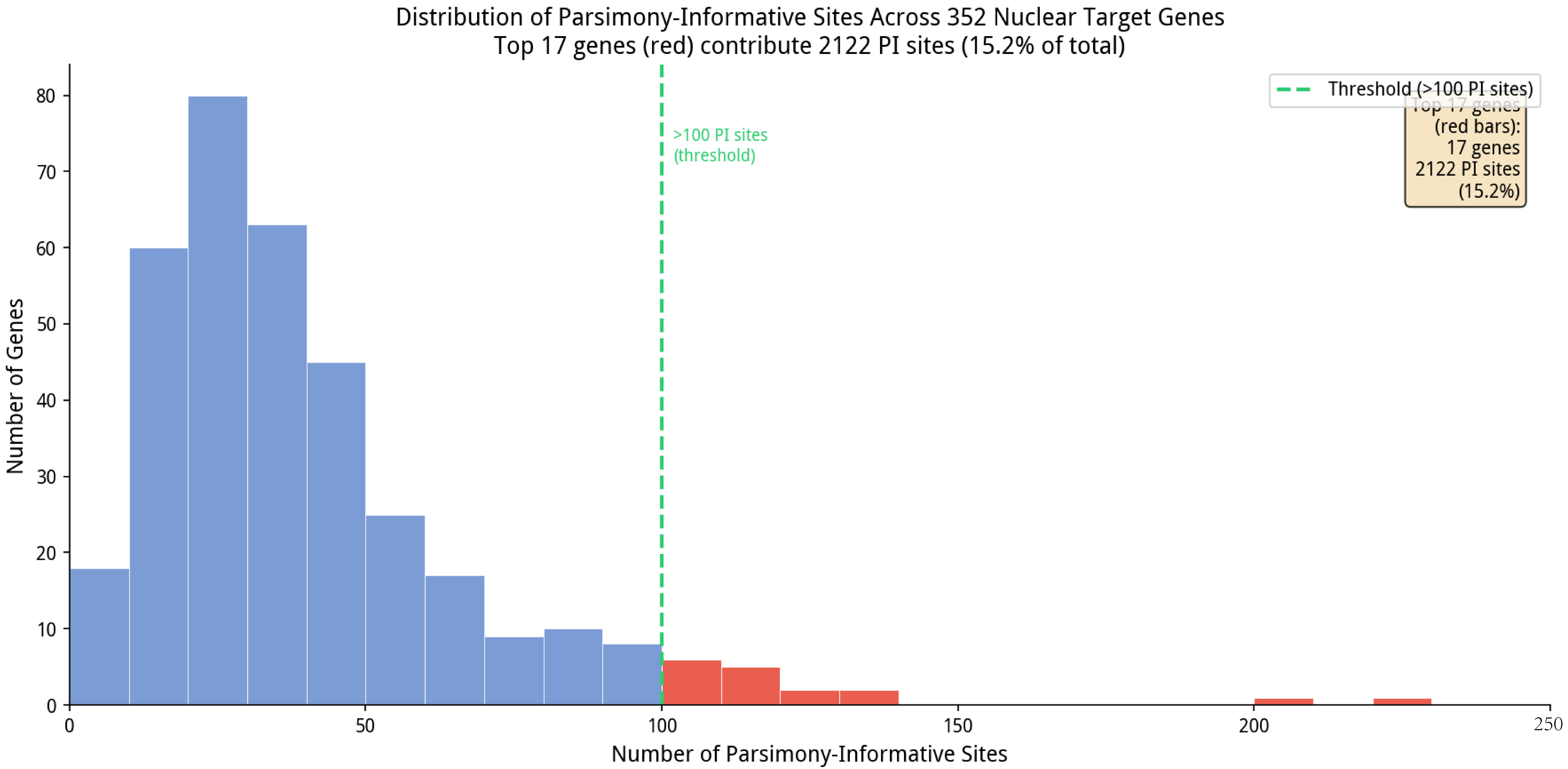

Supplementary Figure S4： Distribution of parsimony-informative (PI) sites across the 352 nuclear target genes. The histogram shows the number of genes (y-axis) binned by their PI site counts (x-axis). Red bars indicate the top 17 genes selected as the core marker set (PI sites > 100), which collectively contribute 2,122 PI sites (15.2% of the total 13,960 PI sites). The dashed line indicates the selection threshold of 100 PI sites.
